# The home bias and the local bias: A survey

**DOI:** 10.1007/s11301-020-00203-8

**Published:** 2020-11-12

**Authors:** Eduard Gaar, David Scherer, Dirk Schiereck

**Affiliations:** Department of Business Administration, Economics and Law, Technical University of Darmstadt, Hochschulstraße 1, 64289 Darmstadt, Germany

**Keywords:** Home bias, Local bias, Domestic bias, Behavioural bias, Market imperfections, D14, D91, E71, G11, G12, G41

## Abstract

The home bias like the disposition effect is a well-researched economic phenomenon in investor behaviour which has been examined in finance journal articles for decades. While there is little doubt about the existence of the bias, its magnitude varies across countries and investor groups. The home bias has to be regarded as a multifactorial phenomenon, a combination of numerous causes which all synergistically contribute. In contrast to other biases the home bias can at least partially be explained by reasons beyond irrational investor behaviour. While institutional restrictions play a minor role, informational asymmetries and superior information of domestic investors are important factors. Thus, the performance of investments may well benefit from a home bias, and the bias then no longer would be a puzzle but rather rational behaviour as a lower diversification level may lead to higher returns. The contemporary understanding of the home bias gains in relevance as the ongoing political debate in Germany has to clarify an institutional framework for long-run retirement savings plans of private households based on equity investments.

## Introduction

In a frictionless perfect global capital market, investors should invest the risky part of their savings completely in the market portfolio to optimize their risk-return patterns and to comply with classical approaches as the CAPM (Sharpe 1964). However, empirical research provides evidence for decades that in real markets investors deviate from this portfolio structure which is optimal in perfect markets. The home bias belongs to the puzzles in economics, proven empirically, that do not fit into (neoclassical) theory (Obstfeld and Rogoff 2000). Following the home bias and the intra-national local bias investors are inclined to invest disproportionately into local and domestic assets, not following portfolio diversification strategies.

Based on capital market models, the home bias has been elaborated (Black 1974; Michaelides 2003; Stulz 1981a, 1981b), and empirical research on the bias started with French and Poterba (1991) studying U.S., UK and Japan. Cooper and Kaplanis (1994) and Fidora et al. (2007) among others confirmed the patterns of the home bias for these three countries. Additional evidence for Germany, France, Italy and Sweden documents the global reach of the phenomenon (Anderson et al. 2011; Chan et al. 2005; Lau et al. 2010; Mishra 2015; Lippi 2016). The local bias as the intra-national equivalent has been detected for intra-U.S. investments (Hong et al. 2005; Huberman 2001), Finish investors (Grinblatt and Keloharju 2001), Japanese investors (Kang and Stulz 1997) and German individual investors (Baltzer et al. 2013). The overwhelming majority of research examines home and local bias dealing with direct equity investments and indirect equity mutual fund investments, but the home bias also can be determined for bonds (Ferreira and Miguel 2011; Solnik and Zuo 2016), real estate (Eichholtz et al. 2001; Imazeki and Gallimore 2009) venture capital investments (Cumming and Dai 2010) and bank loans (Presbitero et al. 2014).

Home and local bias are prevalent and existing for both individual household investors and sophisticated, professional investors like mutual fund managers (Shapira and Venezia 2001) or occupational pension fund managers (Lippi 2016). However, individuals tend to exhibit a higher degree of the bias (Ivkovic and Weisbenner 2005; Lütje and Menkhoff 2007). Most empirical research tried to detect reasons why investors show a home and local bias. It can be discussed if institutional reasons urge investors to show a home bias. Taxes, transaction costs and barriers to international investments may contribute (Black 1974; Michaelides 2003), though institutional reasons are often challenged in literature (Coën 2001; Glassman and Riddick 2001). Informational asymmetries between investors presumably cause home bias. These informational reasons can be seen in a universal setting as general information asymmetries (Coval and Moskowitz 1999, 2001; Dziuda and Mondria 2012; Shukla and van Inwegen 1995) or as informational advantages resulting from different accounting standards between countries (Ahearne et al. 2004; Bradshaw et al. 2004; Eichler 2012). Familiarity also may induce informational advantages, thus a home and local bias (Bodnaruk 2009; Massa and Simonov 2006).

A third, quite popular category in studies are behavioural reasons and home bias then should be related to emotional biases of investments and asset allocation. A general optimism and a strong belief in domestic/local assets (Li 2004; Solnik and Zuo 2016; Strong and Xu 2003) are part of these explanations as well as ambiguity aversion (Dimmock et al. 2016), perceived competence (Abreu et al. 2011; Kilka and Weber 2000) and experience of investors (Graham et al. 2009; Lütje and Menkhoff 2007). Financial literacy and advice seeking of investors (Calcagno and Monticone 2015; Kramer 2016; Mietzner and Molterer 2018) can be assumed being related to home and local bias. ‘Pure familiarity’, a notion coined by Massa and Simonov (2006), and also studied for the local bias by Grinblatt and Keloharju (2001), plays a role as well. Patriotism (Morse and Shive 2011) and loyalty (Cohen 2009) are similar behavioural traits. Hedging motives constitute the last part of behavioural reasons. By exhibiting a home bias, investors may be capable of hedging against uncertainty (generally examined by Choi et al. (2017)) which often takes the form of inflation risk, exchange rate risk and consequential deviations from purchasing power parity (Fidora et al. 2007; Harms et al. 2015). The importance and practical relevance of hedging motives is put into question (Cooper and Kaplanis 1994; Glassman and Riddick 2001; Uppal 1993).

There are many reviews about home bias (Lewis 1999; Wolf 2000; Coeurdacier and Rey 2013; Ardalan 2019). The reviews of Lewis (1999) and Wolf (2000) are dated back two decades and often examined home bias in consumption as well. Meanwhile the number of exchange listed companies dropped significantly in all major stock markets leaving investors with a smaller number of assets to diversify. The sources to collect information about stocks for investments increased with the number of Internet users facilitating the basis for knowledge about foreign assets. And in recent years, the necessity to privately save and invest for retirement purposes became more and more obvious to private households in countries like Germany with a long history of predominant savings only based on bank accounts and life insurances. These changes in the overall institutional environment lead us to derive two research questions for the following literature survey:

1. Is there still convincing evidence for a home bias and a local bias in developed stock markets?
2. If there still is a bias, does this bias result in inferior risk-return structures of private portfolios?

In the case, that both questions have to be answered positively then financial advisors in banks but also the new robo advisors should address this issue to support investors in overcoming the otherwise biased investment behaviour. Compared to the more recent reviews by Coeurdacier and Rey (2013) and Ardalan (2019) the contribution to research of this review can be defined from two sides. First, the studies and papers incorporated in this review differ significantly from the two mentioned and a large part has not been included in the previous reviews. This is partly due to the method of literature selection and partly based on the fact that mainly studies explicitly elaborating home bias are included in this review. Second, the approach of this review is more comprehensive and grasps the broad picture of home bias, especially referring to Ardalan (2019). Whereas Ardalan (2019) exclusively reviews the reasons of home bias encyclopaedically, this paper reviews various measurements, the reasons and implications of home bias. On top of that, the study differentiates between home bias and local bias and simultaneously integrates both.

The paper is structured as follows. First, in Sect. 2 the methodical approach will be explained and how relevant literature has been found and evaluated. Section 3 deals with the precise definition of home and local bias. Section 4 gives empirical evidence on both the existence and the degree of the home bias for numerous countries addressing the first research question. Section 5 constitutes the main body of the review and works out reasons for and consequences of the home and local bias, divided into institutional, informational and behavioural reasons and thereby addressing the second research question. In addition, the implications of home and local bias, especially on portfolio performance, are presented in Sect. 6. Section 7 summarizes the findings and provides avenues for future research.

## Literature selection

The search request started with the identification of the relevant keywords. Although the basic puzzle is called home bias equity puzzle, it is expected that merely the notion home bias is being used in most of the relevant research articles. Besides, there may be several synonyms like local bias or domestic bias. These similar terms are also used for search requests. In a second step, the search is divided into advanced (title, abstract) search and simple full-text-search.

In this review, concerning the considered media type the focal point will be on highly recommended journals in order to concentrate on the most qualified results for the two research questions.^1^ Besides the standard library catalogue, Google Scholar is used as source. The decision falls on Google Scholar as it offers a high number of sources and the goal of the review is to capture all studies on home bias. The results in this review would not change significantly using another bibliometric database. The titles of all identified articles are scanned. However, the findings obtained by the advanced search are subject to a more thorough analysis. Nearly all of these are significant to the researched topic and are included in this review. The number of results in Google Scholar reinforces that the home bias is a broadly discussed topic in finance; just the advanced search findings and not all of the full-text findings with Google Scholar are scanned for relevance.

Apart from actively conducting search requests, the *references* and citations in the articles found by using the two methods are analysed. Relevant papers are drawn from the references and included in the review. This process may be repeated several times. Note, that the quality of every identified paper is evaluated. If the paper, respectively the correspondent journal, has a poor ranking, it will not be included in this review. This cross-reference method is one of the most promising ones in order to find further articles related to the topic. Table 3 in the appendix gives an overview about the results of the home bias search per journal and database.

Following the advice of Fisch and Block (2018) that the screening criteria can have crucial implications for the results, this review solely concentrates on articles published in high quality journals. All types of grey literature like working papers and discussion papers are not incorporated. The evaluation and the selection process is conducted as a combination of objective and subjective methods. The VHB-ranking for a subjective evaluation whereas the impact factors constitute the objective method. The VHB-Ranking is made by skilled and experienced university professors who give their opinion about the quality of journals (The ranking goes from A+ for a worldwide leading journal over A, B and C to D). Even though this seems to be and even is a subjective^2^ judgement and ranking, the VHB-ranking is one of the most qualified rankings one can obtain for evaluating the scientific quality of journals. First, all journals are included which are found in the VHB-ranking and not ranked below B-level. Within this range, higher-ranked journals are preferred over lower-ranked. Second, two different impact factors are used to evaluate the significance of journals. Only if the journal is ranked in the upper half of both considered impact factor surveys, the correspondent article may be included in this review. All other journals and related papers are excluded and neither used, nor cited. The first impact factor is based on the database IDEAS (IDEAS 2019). The impact factor survey “https://ideas.repec.org/top/top.journals.simple.html” is obtained on the Internet. In this survey 2,244 journals (without working papers) are ranked. There are two journals not ranked in the VHB-ranking and not being within the upper half of the IDEAS impact factor survey: ‘Jahrbuch für Wirtschaftsgeschichte’ and ‘International Journal of Financial Research’. These two journals are therefore excluded from this review.^3^ All other considered journals are within rank 707 or above. The second impact factor is developed by ‘Scimago’. There are two considered survey categories of the Scimago ranking. In the category ‘Economics, Econometrics and Finance’ 1,035 journals are ranked (Scimago 2019). The only journal that is ranked below the upper half of the rated journals is the ‘Review of Derivatives Research’. Since this journal is ‘A’-ranked in the prioritized VHB-ranking, it is nevertheless used in this review.

On the whole, applying the evaluation methods, there are two journals not listed: ‘International Research Journal of Finance and Economics’ and ‘Journal of Psychology and Financial Markets’. Thus, the ‘International Research Journal of Finance and Economics’ is excluded from this review. The ‘Journal of Psychology and Financial Markets’ is not included in any rating because it no longer exists under this name. The current name (from 2003 on) of the journal is ‘Journal of Behavioural Finance’ (Taylor and Francis Online 2019). This journal is ‘B’-rated in the VHB-ranking and ranked 320 in the 1,035 journals in the Scimago impact factor survey ‘Economics, Econometrics and Finance’. Therefore, the journal is part of this literature review.

## Defining home and local bias

In general, the home bias describes an investment behaviour in portfolio management where investors tend to overweight their home country’s market and thus investing disproportionally more in assets of their home country compared to its share in the overall market portfolio. In principle, the notion domestic bias is being used with the same meaning.

In contrast, the local bias is more of an intra-national phenomenon. It is always related to distance within a certain country. Investors are inclined to invest a disproportionate high percentage in assets of firms located close to them, independent from country borders. This also results in a lack of diversification. It is also called ‘home bias at home’.

Since the effects and causes of home/domestic bias and local bias are quite similar and both even considered in several papers, the literature on local bias’ effects is fully included in this review. Baltzer et al. (2013) examine the bridge between local bias and home bias. Studying the data of stockholdings from Germany and its neighbour countries they find that the local bias is not limited to national borders, it is cross-border-related. If the investment proximity is close enough, the inclination to local stocks exceeds borders. Therefore, it is possible to perceive foreign countries as local if they are ‘close enough’.

There are some other inclinations towards the domestic market respectively biases in general that are closely related to home and local bias. These are foreign bias observed by Chan et al. (2005), foreign industry bias (Schumacher 2018), home-institution bias (McQueen and Stenkrona 2012), listing home bias (Sarkissian and Schill 2003), flight home effect (Giannetti and Laeven 2012), consumption home bias (Obstfeld and Rogoff 2000) and the academic home bias (Karolyi 2016).

## Empirical evidence and measures for home and local bias

There is much evidence of the existence of home bias and local bias. The degree of home bias can be measured by various approaches for numerous countries. There is an observable variation in the extent of home bias in the course of time. Besides, induced by some factors the degree varies as well.

### Empirical evidence and measures on the existence of home bias and local bias

Evidence on the existence of the home bias can be observed in most countries worldwide. French and Poterba (1991), Cooper and Kaplanis (1994), Tesar and Werner (1995) report evidence for OECD countries and Stockman and Dellas (1989) and Dziuda and Mondria (2012) for additional countries. Oehler et al. (2008) confirm a significant home bias of German mutual fund investors and even a European home bias. German mutual funds not only hold a more-than-optimal share of German assets but also hold higher-than-optimal weights of other European countries’ assets compared to the world market portfolio. Lütje and Menkhoff (2007) also prove the existence of the home bias specifically for German investors, Dahlquist (2001) for investments in Sweden. Lippi (2016) confirms home bias for Italian professional occupational pension fund managers investing in government securities, corporate bonds and equities.

Relating to different asset types, the existence of a home bias has been underlined for equities (Bradshaw et al. 2004; Diyarbakirlioglu 2011; Tesar and Werner 1995), bonds (Fidora et al. 2007; Solnik and Zuo 2016; Tse 1999; Ferreira and Miguel 2011; Tesar and Werner 1995), real estate (Imazeki and Gallimore 2009; Eichholtz et al. 2001) and mutual funds (Coval and Moskowitz 1999; Giannetti and Laeven 2016; Lütje and Menkhoff 2007; Oehler et al. 2008).

There is also much evidence of the existence of the intra-national local bias. Coval and Moskowitz (1999, 2001) document a local bias for mutual funds. Amongst others, Ivkovic and Weisbenner (2005), Seasholes and Zhu (2010), Huberman (2001) as well as Hong et al. (2005) confirm the existence of a local bias of individual U.S. investors. Grinblatt and Keloharju (2001) prove a significant local bias of Finish investors, Kang and Stulz (1997) for Japanese. For German individual investors, Baltzer et al. (2013) also document a local bias. Parwada (2008) examines the location and portfolio choice of investment start-ups. The degree of start-ups’ local bias is three times higher than the local bias extent reported by Coval and Moskowitz (2001). Pool et al. (2012) show that mutual fund managers in the U.S. overweight their home states where they come from. The degree of local bias is hardly measurable and measurements are even worse to compare to each other because local (or regional) is not a clearly defined area, in particular compared to home bias for which the borders (of a country) are clearly determined.^4^ Additionally, it is hard to determine a comparing, well-diversified portfolio which is crucial to measuring the degree of the bias.

In contrary, the home bias can be measured much better. In their basic work on the home bias, French and Poterba (1991) show the degree of home bias for three countries based on data from the end of 1989. U.S. investors invest 93.8% domestically, Japanese 98.11% and UK investors just 82% of their equity portfolios. The lower level of British domestic investments was due to “Prime Minister Thatcher’s relaxation of capital control” (French and Poterba 1991, p. 223). These figures underline that there is a home bias, but the figures do not take into account the optimal weight of every country in a well-diversified portfolio. Also with data from the end of the 1980s, Cooper and Kaplanis (1994, p. 46) support the results of French and Poterba (1991) but calculate a better comparable measure of the home bias by calculating the “domestic equities relative to the proportion of domestic equities in the world market portfolio”: US 98%, UK 78.5%, Japan 86.7%, Germany 75.4%, France 64.4% and Sweden even 100%. Still, the home bias remains significant and strong.

Nowadays,^5^ home bias is measured with numerous approaches (some just slightly different to others). Table 1 gives an overview about the results of home bias measurements of selected countries which are explained briefly in the following section. One way to measure it, is to set the share of domestic assets in relation to the ‘optimal’ CAPM share of domestic assets. The difference of weights of domestic assets (in the actual and ‘optimal’ portfolio) is then considered as a measure of home bias. The CAPM home bias is defined according to Morse and Shive (2011, p. 418):

$$CAPM\ Home\ Bias\; \% = domestic\ holdings\; \%-\frac{home\ capitalization}{world\ capitalization}$$

**Table 1:** Different measurements of home bias for selected countries

	French and Poterba (1991)	Cooper and Kaplanis (1994)	Chan et al. (2005)	Lau et al. (2010)	Own calculation 1	Own calculation 2	Fidora et al. (2007)	Hau and Rey (2008)	Anderson et al. (2011)	Mondria and Wu (2010)	Mishra (2015)
Year of data	1989	1987	1999–2000	1998–2007	1999–2000	1999–2000	2001–2003	2001–2002	2006	1988–2004	2011
U.S.	93.8	98.0	0.61	0.695	0.730	0.3881	75.1	92.1	42.21	0.82	0.6118
UK	82.00	78.5	1.67	1.714	0.380	0.3493	67.1	65.4	20.75	0.73	0.5629
Japan	98.11	86.7	1.86	2.363	0.682	0.6053	89.3	–	14.83	0.91	0.7916
Germany	–	75.4	2.12	2.171	0.307	0.295	61.6	55.4	16.40	0.7	0.4609
France	–	64.4	2.55	2.651	0.533	0.5095	72.4	55.4	32.42	0.82	0.6345
Italy	–	91.0	2.77	3.028	0.339	0.3315	57.3	55.4	16.63	0.83	0.3902
Sweden	–	100.0	3.81	3.927	0.472	0.4571	–	–	41.43	0.77	0.5035

The ‘own calculations’ are based on the data of Chan et al. (2005). ‘Own calculation 1’ is calculated according to Fidora et al. (2007). ‘Own calculation 2’ is the domestic capital market weight subtracted from the share of domestic investments. For Mishra (2015), the measures with the international capital asset pricing model (ICAPM, weekly) are included in the table

Hau and Rey (2008, p. 335) “estimate total investment in the domestic market by domestic agents, … then simply divide it by total domestic market capitalization”. The data is not normalized by the relation of the domestic capitalization to the world capitalization. Fidora et al. (2007) give comprehensive data on the degree of home bias which in contrast to Hau and Rey (2008) is related to the share of the world capitalization based on the formula:

$$Home\ Bias\ of\ Country\ i=1-\frac{w_i}{w_i^{\ast }}$$

Fidora et al. (2007, p. 635) define *w*_*i*_ as the “share of international assets in the country’s portfolio” and ${w}_i^{\ast }$ as the “market weight of the rest of the world seen from the viewpoint of a given country *i*”. Mature economies (e. g. the U.S., the UK, Germany, Japan etc.) exhibit, on average, a home bias of 67.6%. Emerging economies (e. g. in Asia and Latin America) show a significant higher degree of around 95%.

Chan et al. (2005) apply a resembling measure, but express the home bias as a natural logarithm. Lau et al. (2010) calculate their home bias measure exactly the same way as Chan et al. (2005). Since the measurement of Lau et al. (2010) is based on data over a longer period of time (from 1998 to 2007), they obtain slightly different results. Anderson et al. (2011) approach the calculation similar to the general definition of Chan et al. (2005), but distinct in two important aspects: First, Anderson et al. (2011) perform a subtraction and second they do not express the results logarithmically. Both factors cause the very different and not comparable measures of Chan et al. (2005) and Anderson et al. (2011). Mondria and Wu (2010) use the same definition of home bias as Ahearne et al. (2004). Mondria and Wu (2010) define home bias as ‘one minus the ratio share of “foreign equities in country i’s portfolio” and “the share of foreign equities in the world portfolio” from perspective of country I’. Going more into detail regarding the measurement approaches, there are different ways on how to build up the optimal weight of a country of a portfolio. Mishra (2015) shows different approaches and measures of home bias based on different optimal portfolios. A comprehensive measure of both home bias and foreign bias can be found in Cooper et al. (2018) who integrate home and foreign bias in one model and then measure so-called pure home bias relative to the model. Pure home bias is just the part of home bias which cannot be explained by foreign bias and distance effects. Thus, it is not a measure of home bias as considered by the large part of authors (and in this review) and the results are therefore not included in the comparison of home bias measurements in this review. However, the model is very compelling and seems a promising approach different to the large part of existing studies. Cooper et al. (2018) find that pure home bias can just be observed in emerging markets. For developed countries foreign bias can explain the large part of total home bias variation, i. e. “the home country is very much like a foreign country with zero distance. Investors do not appear to exhibit a pure fear of foreign investment separate from their general dislike of distance” (Cooper et al. 2018).

As summarized in Table 1, the measures are quite different. All papers confirm the existence of home bias for all countries although no consistent and standardized measure is applied. That is why the results sometimes show inverse directions. For example, Chan et al. (2005) measure a larger home bias of the UK compared to the U.S., whereas the findings of Fidora et al. (2007) show the contrary, even when neglecting the logarithmic presentation and even though the data is about a similar period of time. The consistency and accuracy of data and measurements is only guaranteed within one specific study and within one specific method of measurement. There are some papers stating that mismeasurement of the home bias leads to its existence. For example, Lewis (1999) states that the used mathematical models may be the only reason that there is a home bias. But, since all of the presented studies provide evidence for the existence with different models, the effect of mismeasurement when dealing with the pure existence seems to be marginal.

### Empirical measuring the varying degree of home bias and local bias

The degree of home bias varies by two points of view, a general decline in home bias in the course of time and relating to particular factors which impact the extent of home bias. Since most of the available data is provided to U.S. investors (Karolyi 2016), the degree of home bias for U.S. investors is best analysed in empirical research (Eichler 2012). For local bias, there is no disposable data.

Since the percentage of foreign ownership at the Japanese stock market from the 1970s to the 1990s has increased, it can be considered as an indication of a general decline of home bias on course of time (Kang and Stulz 1997). Explicitly measured decline by Levy and Levy (2014) shows a decrease of U.S. home bias from 1988 until the 2000s. This finding is confirmed by Ahearne et al. (2004). After the early 2000s, home bias first slightly increased but has fallen again until 2012 and remains on a significant level around 40%. Support of the decline of home bias for other countries comes from Fidora et al. (2007). Both equity and bond home bias in mature markets have decreased during 1997 to 2003. Unfortunately, more recent data on the degree of home bias for other countries than the U.S. could not be found in any considered paper.

The home bias also varies due to particular factors/variables. The economic respectively financial development of a country is one factor, however most studies show that there is no statistically significant correlation and impact. Bae et al. (2008) exclude economic development of a country as a driving force for the equity home bias. Also, Dahlquist et al. (2003) challenge the influence and importance of the financial development on the equity home bias, as differences in financial development will be reflected in stock prices. Chan et al. (2005) support these findings and do not find a significant impact of economic development. They detect that merely the stock market development and familiarity have a statistically significant influence on the extent of home bias. Imazeki and Gallimore (2009) use the same approach as Chan et al. (2005) but examine real estate mutual funds and report similar results. The only significant factor which influences the degree of home bias in real estate is a combination of two variables: real estate market capitalization size and real estate market transparency. According to Imazeki and Gallimore (2009) general economic development also seems to not be important when studying real estate home bias. However, there is evidence for an influence of the variable ‘economic development’ with respect to home bias in bonds (Ferreira and Miguel 2011).

In accordance with Pool et al. (2012) resource-constraints of managers influence the degree of home bias. Managers with more limited resources exhibit more home bias. Investors with a small amount of invested money are more inclined to exhibit home bias (Karlsson and Nordén 2007). The effect of the size can be also transferred to the countries’ size, i.e. the size has a positive impact on home bias as in a big country an investor has more opportunities to diversify his portfolio and is not dependent on diversifying internationally (Mishra 2015).

Anderson et al. (2011) examine the influence of culture on home bias and show that high values of the variables long-term orientation and masculinity lead to a relative decrease in the level of home bias, whereas uncertainty avoidance as a cultural characteristic increases home bias. The influence of gender is also proven by Karlsson and Nordén (2007) who show that overconfident investors (mostly men) are more probable to show home bias. Lütje and Menkhoff (2007) also underline the influence of overconfidence on home bias.^6^ The impact of cultural variables on investment decisions is confirmed by Beugelsdijk and Frijns (2010), though only for foreign bias.

Employees in the public sector (having a high job security) acting as investors and investors with a low education/sophistication are more inclined of being home-biased (Karlsson and Nordén 2007). According to Mondria and Wu (2010) home bias decreases with financial openness but remains in the long run due to interaction between “portfolio and information choices” (Mondria and Wu 2010). Banks just like institutional ‘investors’ also exhibit an information-based home bias when they give loans to enterprises (Presbitero et al. 2014). Banks even exhibit home bias when allocating their own bank assets and do not diversify internationally with the help of international subsidiaries (García-Herrero and Vázquez 2013).

Shapira and Venezia (2001) examine differences in (behavioural) patterns of institutional and individual investors and show that professional investors have a better diversified portfolio (less home bias) than individual investors. Sometimes the individual investors influence the institutional one. That means that the institutional investor is the one who actually invests but has to consider the ‘wishes’ of the individuals, e. g. considering mutual fund investing (Oehler et al. 2008). Lütje and Menkhoff (2007) for home bias and Ivkovic and Weisbenner (2005) for local bias provide similar evidence in favour of a higher bias of individual investors.

## Reasons and causes of home bias and local bias

The reasons for a home bias and local bias may be divided according to French and Poterba (1991) into two types: institutional and individual investor-related reasons. Nowadays, since research has advanced, it seems appropriate to add one further reason, i. e. information. Information-based explanations constitute a big part of recent research and the large part of recently published articles. Nearly all of the literature discussed in this section refers to home and local bias for volatile assets which are equities/stocks and mutual funds investing in stocks.

### Institutional reasons

In their early work French and Poterba (1991) mention institutional aspects such as capital flow restrictions, taxes and transaction costs as reasons for home bias. However, they do not find significant evidence for these reasons and conclude that institutional reasons may account for a certain degree of the home bias, but are unable to explain the large extent. In recent research the relevance of such institutional reasons is declining or the reasons are even rejected. Institutional reasons consist of reasons which the actual investor cannot influence and which are set up by policy makers or are based on general economic principles. Institutional reasons cannot explain any local bias. The identified reasons just relate to international home bias.

#### Transaction barriers and taxes

Transaction barriers are explicit barriers to an investment abroad and can be induced by different causes: taxes on foreign investments or not further specified transaction costs (e.g. cost for opening an investment account abroad). In the early 1970s, Black (1974) develops a model with a capital market equilibrium combined with explicit barriers (taxes) to foreign investment. In his model, a home bias occurs with these taxes on foreign investments. However, the results are not validated empirically. Stulz (1981b) constructs a similar model by introducing barriers to international investment. Compared to Black (1974) the model has slight differences in the assumptions, especially in the calculation of the costs respectively taxes. Though, he finds similar results that holding foreign assets is costly to domestic investors, therefore hold less by domestic investors. Stulz (1981b) also gives no empirical support to his hypotheses and to the predictions he derived from his model. But, even in a model without barriers to international investments, investors hold a higher share of domestic assets than expected by standard portfolio theory (Stulz 1981a). The result can be considered as a first step of doubting explicit barriers to investment as an explanation for home bias.

In order to assume transaction costs as a reason, risk aversion levels have to be set to unreasonable extents that cannot be shown in data (Cooper and Kaplanis 1994). Tesar and Werner (1995) explain that the turnover rates for foreign stocks are higher than for domestic stocks. Given that fact, transaction barriers and costs cannot be considered as a plausible explanation. Also, French and Poterba (1991) support the hypothesis that taxes are possibly not the reason for home bias. In a more recent reconsideration of Tesar and Werner (1995), Warnock (2002) approves the basic finding: transaction costs are not able to explain home bias.

Model-based, Michaelides (2003) shows that small additional costs to foreign investments can generate a home bias. These costs may result from fees to international investment or costs of opening a foreign account. Michaelides also suggests information asymmetry as a reason. Very high taxes and cross border taxation may lead to home bias (Mishra and Ratti 2013). However, such high tax rates do not seem to be existent in today’s economy. An appropriate treatment by countries’ policy makers with double taxation is important.

As there has been more deregulation and liberalization of capital markets and capital flows over the last decades, home bias should have been decreased significantly which is not the case. Recent studies challenge explicit barriers as an important reason. Ahearne et al. (2004) test the impact of direct and explicit barriers on international investment. They find that even though these barriers are statistically significant they are not economically meaningful. Dahlquist et al. (2003), Coën (2001), Glassman and Riddick (2001) and Baltzer et al. (2013) challenge the explanations of home bias induced by explicit barriers as well. None of the studies directly rejects any influence of transaction costs on home bias at all, it is rather that transaction costs and taxes as the single reason are unable to fully explain home bias. There is support of the fact that direct costs on foreign investments exist and contribute to a certain, however undefined, degree of home bias. This seems plausible since there are still some kind of cross-border transaction costs in most countries.

#### Correlation between markets

Costs for foreign investments have decreased enormously in the last decades (Levy and Levy 2014). The existence of costs on foreign investments is confirmed, but these costs do not serve as an explanation of home bias. Due to the decrease in general foreign investment costs the home bias should have been decreased as well. Correlation of markets leads to extra costs for investors. Since correlation of markets has increased significantly, the sum of the costs remains stable and thus home bias as well (Levy and Levy 2014). The induced additional cost is proportional to the so-called ‘home bias magnification’ (HBM) factor that can be calculated with the following formula, “where *ρ* is the average correlation between markets” (Levy and Levy 2014):

$$HBM=\frac{\rho }{1-\rho }$$

The HBM explains the finding that there is no economic benefit from investing abroad in highly correlated markets. In summary, high correlation equals low diversification gains. Based on a model, Michaelides (2003) also supports the idea that a positive and strong correlation between domestic and foreign markets leads to a significant home bias.

#### Internal governance of firms

Internal governance of firms is seen as an institutional reason for home bias because the investors are not able to change any of these facts. According to Dahlquist et al. (2003), across 51 countries 32% of shares are not traded at all which means that these shares are not available for public investors. They are with controlling shareholders (e.g. a family or similar). In the U.S., the percentage of controlling shareholders is lower. Consequently, investors cannot hold the world market portfolio (which assumes that all stocks are traded) even if they would like to, regardless of and independent on any other reasons for home bias. Home bias is significantly smaller for the U.S. and other countries taking into account the fraction of controlling shareholders. Mishra (2015) measures ‘institutional quality’ which highly influences corporate governance as well. He finds a correlation to home bias and claims that good corporate governance is appreciated and therefore leads to lower home bias.

Poor governance of firms can also be shown in another aspect, meaning high managerial control and a high level of insider control. If a firm is poorly governed, foreigners are inclined to hold fewer equities of such firms, thus investing more in their domestic market, exhibiting home bias (Leuz et al. 2009). In poorly governed firms, expropriation and governance problems are more likely. These results can help to explain home bias. Investors are ‘forced’ to invest disproportionately in their domestic country (exhibit home bias), because the world market portfolio is not available to them for investment.

### Informational reasons

French and Poterba (1991) already mention informational aspects as one possible reason for the shown investor-specific behaviour: “They [investors] may impute extra ‘risk’ to foreign investments because they know less about foreign markets, institutions, and firms” (French and Poterba 1991, p. 223). Different information results in different expected risk-return patterns, hence inducing home bias if the perceived information advantage is towards domestic assets.

#### General informational asymmetries

Information asymmetries are a reason for both intra-national local bias and international home bias. Shukla and van Inwegen (1995) provide the first study on informational advantages as a reason for home bias and show that domestic investors (U.S. investors) have an information advantage compared to foreign investors (UK investors). The prevalent asymmetry in information induces a home bias of the UK investors, because they underweight the foreign U.S. market. The behaviour of underrating the U.S. market seems rational due to the poor performance of foreign UK investors which discourages them from investing abroad. Both Zhou (1998) and Michaelides (2003) in a model and Coval and Moskowitz (1999) confirm the existence of informational asymmetries as an explanation of home bias for investors whereas Presbitero et al. (2014) for banks giving loans.

When dealing with informational asymmetries and home bias, the question arises why informational advantages in a world with a high level of information transmission (especially by the Internet) still exist. van Nieuwerburgh and Veldkamp (2009, 2010) show that the advantages are based on fundamental and natural human behaviour: Information is rated differently depending on the exclusiveness. Exclusive information is worth more. These findings of investors’ behaviour can explain both local and home bias, national borders are not taken into account. So, when taking information-based explanations of home bias it is not just about the existing advantage, it is also about the learning process of obtaining information. This result is closely related to Choi et al. (2017) who conclude that the higher the learning capacity of an investor, the more concentrated the portfolio is. Since home bias is a type of portfolio concentration, this finding means that an investor is inclined to learn more and more about the assets he already knows in order to obtain specific information. The higher the learning capacity the better the behaviour works out.

The model by Dziuda and Mondria (2012) supports the information-based explanation of home bias. But in their model, they attribute the informational advantage to the clients of professional investors (managers). The managers’ reply leads to a reinforcement cycle. Thus, they prove information to be the reason for home bias, however challenge the source of the information asymmetry and advantage as determined in Coval and Moskowitz (2001). These differences can be due to studying intra-national investment and local bias (Coval and Moskowitz 2001), whereas Dziuda and Mondria (2012) exemplify international portfolio choice and home bias. The results of a survey (run in 2003) about German fund managers analysed by Lütje and Menkhoff (2007) are rather in accordance with Coval and Moskowitz (2001) that the existence of home bias is unrelated to clients’ preferences. The fund managers themselves perceive a local information advantage, expect higher returns of domestic investments and therefore their investments are home-biased. This perceived information advantage does not exactly hold to be true. Lütje and Menkhoff (2007) conclude that pure informational explanations of home bias should be challenged.

Hong et al. (2005), in contrast to Coval and Moskowitz (1999, 2001) who study how investors gather information, examine how investors share information. Investments in one city correlate, i. a. by word-of-mouth information transmission between investors. The finding constitutes a reinforcement process of any local bias or home bias, because one investor who is slightly biased transmits this bias to other local investors. It comes to a positive feedback amongst investors located nearby. Hence, information asymmetries play an important role in explaining home bias (Hong et al. 2005). Also, Hau (2001) identifies information asymmetries to be inducing home bias. The discovered information asymmetries are caused by linguistic and cultural differences between traders. A pure geographic bias which is just based on distance and not informational aspects can neither be confirmed nor fully discarded.

The above-explained standard local information approaches claim that there is only local/intra-country information and better knowledge. Albuquerque et al. (2009) introduce ‘global private information’ because local information alone cannot explain different performances of investments with informational advantages.

Local bias is closely related to distance effects meaning geographic proximity. If an investor is located more proximate to a potential investment opportunity, she has often access to more and better information, the accessibility of information is better and less costly. Three firm characteristics (based on U.S. data) lead to an informational advantage and thus to local bias: a small firm size, a high leverage and a low international output tradability (Coval and Moskowitz 1999). If all of these three characteristics are given, there is the biggest informational advantage for local investors. The result can be easily explained, because it is precisely for such characterized firms that local information can be obtained most easily and informational advantages have the biggest impact on performance (e.g. the firm is not known as widespread, therefore the information about the firm is not either). As well, geographic proximity leads to informational advantages for local investors. Local investors have lower costs to monitor the local firm and the local stock or have special access to specific, investment-relevant information (Coval and Moskowitz 2001).

Baik et al. (2010) and Gaspar and Massa (2007) also support the information advantage theory of local investments. Ivkovic and Weisbenner (2005) and Ivkovic et al. (2008) go the argumentation the other way around and conclude from a better performance of a biased/concentrated portfolio as local information advantage. They find that investing locally in combination with a concentration on a very few stocks, the best results would be yielded. Besides, by mimicking the behaviour of the local investors, outside investors are able to increase returns (Ivkovic and Weisbenner 2005).

Information advantages also cause the local bias for investment decisions of start-ups (Parwada 2008). The founders are able to maintain their local and familiar network of the former employment and use the local information. This leads to a local bias in equities three times higher than the local bias of mutual funds, reported by Coval and Moskowitz (2001). Even investment banks exhibit local bias due to informational advantages when placing municipal bonds especially by local investment banks (Butler 2008).

The reason ‘informational advantage’ for local bias is detected for both investor types but unfortunately the evidence is limited to U.S. investors investing in the U.S. Only Bae et al. (2008), on the basis of analysts’ data worldwide, observe an information advantage for local investors respectively analysts over foreigners.

Some studies challenge the information-based explanations of local and home bias. Even though Seasholes and Zhu (2010) state that there is a local bias in the U.S., they do not observe informational advantage of local investors over foreign investors. The same conclusions are made by Pool et al. (2012). Both findings are based on the comparison between the performance of biased and not-biased portfolios. It may be the case that the possibly existing surplus of information of a domestic or local investor has a poor quality, hence not results in higher returns. Glassman and Riddick (2001) subsume information asymmetries to differential perceived riskiness of foreign assets and claim that perceived riskiness adjustments cannot explain home bias solely. Though, all these studies questioning information-based reasons follow rather implicit approaches, i. e. concluding from performance results on possible reasons.

#### Accounting and reporting standards as informational asymmetries

Accounting and reporting standards are a reason mentioned frequently in research about home bias. It is reasonable to assume that the adoption of accounting standards is related to information asymmetries. As Bradshaw et al. (2004, p. 836) say “informational issues that affect home bias are multilevel and at least partially due to reporting decisions”.

Examining investment decisions of institutional U.S. investors in non-U-S. firms,^7^ Bradshaw et al. (2004) document that if a non-U.S. firm has a high level of adoption of U.S. accounting standards, U.S. investors invest more in such a firm. Thus, there is a higher degree of diversification, hence less investment in the domestic market (home bias). The adoption of U.S. accounting standards contributes to a reduction of information processing costs for potential investors. Besides, investors feel familiar and comfortable with the well-known standards. U.S. investors are home-biased towards accounting standards that they know (Bradshaw et al. 2004). With a different approach, Ahearne et al. (2004) confirm that information costs and asymmetry are highly related to accounting standards. Firms are able to reduce information costs for potential investors by listing their equities publicly in foreign indexes and thus have to comply with the regulatory issues. For example, just 18% of German firms are listed in the U.S., while 81% of Dutch firms. That is one potential reason why U.S. investors underweight German companies and assets much more in their portfolio compared to Dutch. If all firms were listed in the U.S., a large extent of home bias should be eliminated. Though, not all of it as public listing and adopting certain accounting standards are only two aspects contributing to home bias, i. e. not being able to entirely explain the extent (Ahearne et al. 2004). Mishra (2015) supports the finding that foreign listing has an negative impact on home bias. Aggarwal et al. (2005) report that U.S. mutual fund investors have certain preferences (factors) related to accounting issues when investing in 30 emerging markets. Country-specific factors consist of accounting standards, shareholder rights and legal framework. The issuance of American Depository Receipts, accounting transparency and the voluntary adoption of accounting standards belong to firm-specific factors. All of the mentioned factors have to be at least partially fulfilled, otherwise home bias occurs for U.S. investors.

There might be different results for other countries since U.S. accounting standards belong for decades to the highest quality standards set (Bradshaw et al. 2004). In other countries with less-qualified standards (e.g. Germany or France), investors may be less biased towards their domestic standards. However, it is expected that accounting standards also play a role for those countries when explaining home bias, though having less importance. Covrig et al. (2007) provide evidence on accounting standards as a reason for home bias in an international setting. Using International Accounting Standards (IAS), firms are able to attract more foreign investors, because IAS contribute to better and more useful information for those foreign investors. Therefore, the degree of home bias for foreign investors declines. For firms operating in a poor information environment with low visibility the impact is even higher. The number of foreign investors increases (controlled for other variables) from 2% to 2.9% when adopting IAS compared to local accounting standards.

A higher level of corporate disclosure (resulting in more information for potential investors) is able to reduce home bias (Eichler 2012). Two prerequisites make the corporate disclosure efficient to reduce home bias. On the one hand, security laws have to make the statements credible and reliable by punishing false information. On the other hand, the disclosure statements have to be understandable for the investors, i. e. the investor can interpret them easily without much incurred additional ‘cost’ for understanding. In contrast to the above-mentioned studies, which discussed the regulatory requirements for accounting standards (Eichler calls this corporate disclosure *de jure*, from law), Eichler (2012) shows that only the formal regulatory requirements have no impact on home bias. For the investors, it is always of importance how they are applied (the corporate disclosure *de facto*), which means how a specific firm conducts their accountings and disclosure. But the actual *de facto* corporate disclosure is normally highly dependent on the *de jure* regulatory requirements for accounting. Slightly different assumptions when calculating the results and impacts may lead to such different results.

#### Information-based familiarity and information asymmetry

An investor who is familiar with a firm may have informational advantages about the company, so familiarity is in some cases highly related to information asymmetries. This so-called information-based familiarity should not be confused with behavioural familiarity (Massa and Simonov 2006). Investors are inclined to invest in assets which they are familiar with. Massa and Simonov (2006) see familiarity to stocks in the meaning of geographically and professionally close and information-driven. Bodnaruk (2009) supports the existence of familiarity in combination with information asymmetry as a reason for local bias. In a setting of moving, the evolution of familiarity can be observed. Former ties to firms get loose and new ties and familiar relations are build up close to the new residence (Bodnaruk 2009).

The evidence found on information-based familiarity is limited to local bias and both studies are relying on the same data, i.e. Swedish data and investors. Thus, the explanatory power of these specific findings is narrowed. However, without distinguishing information-based familiarity and general information asymmetry but rather considering information-based familiarity as a normal informational reason, the evidence on information-based explanations for home and local bias is still overwhelming.

### Behavioural and individual investor-specific reasons

Behavioural and individual reasons are caused by human’s nature. Behavioural reasons are directly related to investors’ beliefs, perceptions and personality and are normally uncorrelated with market’s development. The introduced behavioural reasons are optimism, ambiguity aversion/competence/experience/financial literacy, pure familiarity/patriotism/loyalty and hedging against uncertainty. These reasons for home and local bias constitute, combined with the informational reasons, the major part of explanations.

#### Optimism and beliefs towards domestic assets

Investors are, in general, more optimistic about the domestic market and systematically assume higher expected returns. This perception results in biased portfolio choices, thus providing an explanation for home and local bias. First, two types of optimism, absolute optimism and relative optimism, have to be distinguished. According to Strong and Xu (2003, p. 308), absolute optimism “occurs when investors are more optimistic about their home market than they are about foreign” whereas “relative optimism towards domestic equities occurs when investors are more optimistic about their home market than are investors from other countries”. Both types of optimism contribute to the explanation of home bias.

French and Poterba (1991) already suggest that investors systematically are more optimistic about the domestic market. Shiller et al. (1996) study the expectations, representing the degree of optimism, from Japanese and U.S. investors based on empirical data and surveys. The answers in the survey correlate, but there is a vast difference in the actual numbers. The investors always exhibit a higher relative optimism for their domestic market which helps explaining home bias. Shiller et al. (1996) are aware of possible information asymmetries, but assume that both investors have nearly equivalent information.

In a model with a standard Bayesian approach, higher expectations of investors for the domestic market are confirmed (Pástor 2000). Prior beliefs are incorporated in the model. Therefore, it is possible to be free from the two disputing approaches: relying fully on standard asset pricing models or not believing these models at all (just relying on data). Concluding from the model, home bias can be justified for U.S. investors when the prior beliefs are stable in reality. Though, Pástor (2000) gives no empirical evidence of his conclusion. Li (2004) puts empirical evidence on the model of Pástor (2000) setting the parameters of prior beliefs consistent with existing literature. In this framework, when computed with actual G7 data, Li (2004) supports the hypothesis of Pástor (2000). Investors consider foreign investments much riskier, resulting in a higher expectation and a higher optimism for the domestic market.

Based on a survey of fund managers, there is further and more comprehensive evidence on differences in optimism as an explanation for home bias. The fund managers surveyed exhibit a higher relative optimism towards the domestic markets (Strong and Xu 2003). The evidence on absolute optimism is not directly supporting home bias. Only for European and Japanese fund managers, an absolute optimism can be found, hence explaining home bias. However, according to Strong and Xu (2003), the absolute optimism findings are subject to the studied time period. In summary, fund managers have “a bias towards domestic equities and a relative bias against foreign equities” (Strong and Xu 2003, p. 312). The result that relative optimism has a positive relation to home bias in portfolio holdings of equity and bonds is confirmed by Solnik and Zuo (2016). They are the first to give evidence on a broader time span which is independent of market phases (bull, bear, market crash).

For specifically German equity fund managers, a relative return optimism for domestic securities leading to home bias towards German investments can be found (Lütje and Menkhoff 2007). Lai and Teo (2008) discover that local analysts of eight Asian emerging countries are more optimistic about the domestic market than recommendations from foreign analysts. If potential investors base their investment decision upon the recommendations of local analysts, this finding can explain home bias.

As a result, there is much evidence of relative optimism for numerous investors’ countries. However, the studies on absolute optimism are limited. Relative optimism should be considered as an important explanation contributing to home bias. Prior beliefs contribute to a certain degree to the existence of home bias. In general, home bias in equities and bonds is driven by behavioural and informational factors whereas home bias in bonds is also influenced by institutional factors (e.g. capital control, investor protection, legal framework). Though, there may be some limitations to the evidence that should not be neglected. Strong and Xu (2003) question what came first and what results: relative optimism or home bias. At least mutual dependencies can be confirmed. But, behavioural reasons, even for sophisticated fund managers, cannot be rejected. Informational reasons together with behavioural causes can exist simultaneously and both contribute to home bias (Lütje and Menkhoff 2007).

#### Ambiguity aversion, competence, experience and financial literacy of investors

In decisions under uncertainty individuals’ preferences are numerous. Ambiguity and aversion to ambiguity of individual investors are one inclination of investors’ behaviours. Some behaviours cannot be explained by ambiguity aversion, but rather based on competence and experience of investors. Besides, financial literacy and the extent of advices seeking are other behavioural approaches.

Ambiguity aversion is a behavioural characteristic of human beings and was tested and confirmed in an experiment by Ellsberg (1961). In an ambiguous investment setting no return distributions are known at all. These findings are “in trouble with the Savage axioms” (Ellsberg 1961, p. 651). Relating to portfolio choices of investors, ambiguity aversion can be part of an explanation for home bias. In a recent study, Dimmock et al. (2016) examine how ambiguity aversion refrains investors from investing abroad. Ambiguity aversion is negatively correlated to foreign stock ownership. This means that ambiguity-averse household investors hold less foreign equities in their portfolio than an average investor. Since the majority of people is ambiguity-averse, there is a certain degree of home bias. Guidolin and Liu (2016) provide supporting evidence in line with the results by Dimmock et al. (2016) based on a model with incorporated prior beliefs of an U.S. investor in the domestic CAPM. In contrast to Pástor (2000), they do not rely on the standard Bayesian approach. Within this model, ambiguity aversion of an investor leads to strong and significant home bias in both bull and bear periods. It is independent from the extent of risk aversion and from the degree of prior beliefs respectively trust about the efficiency of the domestic CAPM. Guidolin and Liu (2016) validate the model based on empirical data. According to Giannetti and Laeven (2012, 2016) ambiguity aversion is reinforced in times of crisis. Giannetti and Laeven (2016) are the only assuming that amongst other reasons ambiguity aversion intensifies local bias (and not home bias) of investors in times of crisis.

Beyond the probability-based (ambiguity) explanation, Heath and Tversky (1991) examine the rather just psychological aspects of the preference for competence-based decisions. The preference of investors, that they establish, is called ‘competence hypothesis’.^8^ This theory is highly applicable to investors’ portfolio choice and home bias. The event of being competent and skilful in this setting is like betting on—in this sense meaning investing in—domestic assets. First, it is, in fact, the case that investors perceive themselves as more competent about assessing domestic assets and overestimate their own judgements. Second, when investing in domestic assets it is generally assumed that the investor has to be more competent. The investor would have the ability to gather information and would have easier access to knowledge about the investment opportunity.

Kilka and Weber (2000) examine the perceived competence of investors and the implications on the expected returns in an upswing market and conclude that competence-based asymmetric judgements and expected returns of domestic stocks compared to foreign stocks contribute to the existence of home bias. Individuals feel more competent about assessing domestic stocks and perceive them more valuable for making investments.

The feeling of competence is closely related to experience. Making and gathering experience can lead to a higher perceived competence of investors. Abreu et al. (2011) show that investors want to acquire experience by investing in domestic securities and consider their first step to investing abroad thoroughly. Investors who invest domestically more often tend to decide on investments abroad earlier, because, based on the domestic experience, they feel more competent about investments in general, including foreign investments. Married, female and older investors wait longer until their first investment abroad; wealthier investors and better educated investors start earlier. According to Graham et al. (2009) especially male investors, investors with a larger portfolio and with more/better education feel more competent. These investors with a higher perceived competence exhibit less home bias.

Overall, it can be concluded that a learning process is an important feature of foreign investment. This learning process has, in general, similarities to the learning process examined by van Nieuwerburgh and Veldkamp (2009), though the two findings have to be distinguished. For both studies learning is important for portfolio choice. However, van Nieuwerburgh and Veldkamp (2009) study learning and information which always results in an information advantage. Abreu et al. (2011) use a more general setting about learning and competence perception, no matter whether there are any competence-based informational advantages which may induce a better performance. This difference demonstrates the importance of why we distinguish between behavioural respectively personal reasons and information-based reasons for home bias.

Some other studies merely examine the experience of investors. Measuring experience by the age variable (high age is equivalent to a high experience level) the degree of home bias decreases with the age of the investor (Lehmann-Hasemeyer and Neumayer 2019). This result is contrary to Lütje and Menkhoff (2007) who consider the age as a determinant of risk aversion and thus conclude that home bias increases with advancing age. They do not relate age to experience. But, Lütje and Menkhoff (2007) also report that investors with less experience exhibit more home bias. Both Karlsson and Nordén (2007) for home bias and Pool et al. (2012) for local bias document that less experience has a positive impact on the bias. The opposition in the findings about age and home bias cannot be resolved. Presumably, the impact of age on home bias is dependent on the fact whether risk aversion or experience is stronger at influencing home bias.

Closely related to experience and competence is financial literacy which is examined in many studies and might be related to home bias. Financial literacy especially determines the degree of advice seeking of investors. Investing abroad without consulting advisors is generally considered risky. Calcagno and Monticone (2015) point out that financial literate people consult advisors with a higher probability. This means that advisors do not resolve the problem of low financial literate investors who rely on their own competence. Kramer (2016) differentiates between perceived and objective literacy. Investors who are confident about their literacy ‘are less likely to seek financial advice’, however referring to a objective measure no relation can be found. The advice from banks to (in particular illiterate) investors seems not to be the best for fulfilling the individual investors’ goals (Mietzner and Molterer 2018). This statement is mainly due to high commission and bank fees.

Consequently, on the one hand being financial illiterate and lacking of advice may lead to poor diversification, i. e. under-diversification of the portfolio. Home bias is such type of poor diversification. On the other hand, the received advice may not always be beneficial for preventing biased investment decisions. However, these are just assumptions and not empirically fully proven yet. Therefore, financial literacy and resulting advice seeking and their potential influence on home and local bias should be studied in future research. In a single study, von Gaudecker (2015) proves that both financial literate households and those who seek advice show a lower degree of under-diversification and thus their investment outcomes are better. This study can be the starting point for future research by relating under-diversification more detailed to home bias as a specific type of poor diversification.

All in all, there is much evidence that competence and experience do have an impact on home bias. It seems reasonable to argue that investors feel more competent about domestic assets and therefore are home-biased. The influence of experience is obvious and confirmed as well: Less experience leads to a higher degree of home bias. The influence of age is ambiguous, making no conclusion possible. There are no findings about competence and local bias, it may be supposed that the results are also valid for local bias. Lack of experience has a positive influence on local bias. Concerning financial literacy and advice seeking, there is no direct evidence on home and local bias, however a relation can be assumed.

#### Pure familiarity, patriotism and loyalty

Both familiarity, patriotism and loyalty are closely related to each other since they all perceive the bond/solidarity of investors with their country. As Grinblatt and Keloharju (2001) claim, familiarity and patriotism are similar and hard to distinguish. Familiarity can be driven by information advantages and vice versa. Though, familiarity is often examined on its own without any relation to information, labelled ‘pure familiarity’ or behavioural familiarity (Massa and Simonov 2006). Behavioural familiarity of investors can contribute to home and local bias as well, since investors are more familiar with domestic/local assets. The characteristic of pure familiarity is that investors show no performance improvement when investing based on pure familiarity. Better performance would only occur if investors acted according to information advantages and information-based familiarity. Though, different to many studies, in this review not all of the analyses, that state a not-better performance of biased portfolios, are considered to be in favour of the familiarity explanation for home and local bias but have to provide explicit evidence that pure familiarity plays a significant role to explain the bias. Tse (1999) for the bond market supports the pure familiarity hypothesis. McQueen and Stenkrona (2012) consider familiarity as the reason for the so-called home-institution bias, another phenomenon closely related to original home bias. The home-institution bias is the highest for provincial and unsophisticated investors, especially with low education, low income and low trading frequency.

For local bias of Finish investors, Grinblatt and Keloharju (2001) identify pure familiarity as a reason. First, investors prefer to invest in firms which are located nearby. Second, investors select firms with the annual reports in their native language. Third, they invest more money to firms whose CEO has a cultural background the investor is familiar with. The effects are less prevalent for well-versed and sophisticated investors. However, it remains unclear if these results can be transferred to higher capitalized markets like the UK or the U.S. Also, Pool et al. (2012) reject the information-based explanation of local bias and identify pure familiarity as a reason for local bias, even among professional investors in the U.S.. These findings are consistent with Huberman (2001) who documents familiarity as an explanation for investment decisions of individual investors. He defines pure familiarity as “a general sense of comfort with the known” (Huberman 2001, p. 678). According to Giannetti and Laeven (2016), who study local bias, familiarity aspects are probably even higher in an international setting, like home bias. Bhattacharya and Groznik (2008), Loughran and Schultz (2005) and Morse and Shive (2011) also report familiarity as a reason for home and local bias.

In contrast to general pure familiarity, which is rather referring to the individual, patriotism and loyalty are often attributed to a larger and specific group of people. Morse and Shive (2011) examine the influence of patriotism on portfolio choices and home bias. From a survey of 53 countries they find that patriotism and home bias are positively related. In a more patriotic country, an average portfolio consists of more domestic equities compared to a less patriotic. The results are valid and robust for other home bias reasons. Within a country, the level of home bias is also lower for regions with a lower level of patriotism. Cohen (2009) shows that investment decisions in an intra-national setting are driven by loyalty which is similar to patriotism. If the finding of Cohen (2009) can be transferred to international investment decisions and home bias, remains unclear.

#### Uncertainty and hedging against uncertainty

Human beings try to avoid uncertainty or at least want to reduce the impact of uncertainty. In a financial setting, an instrument of trying to control uncertainty is called hedging. In principle, hedging can be regarded as a reason which is based amongst others on the individuals’ risk aversion. The circumstances and the framework why investors hedge (real exchange rate volatility and inflation risk) are given in economy and therefore hedging could also be considered as an institutional reason. But, since the focus is on the individual herself, hedging is here subsumed to behavioural aspects.

Choi et al. (2017) show that the extent of home bias and uncertainty in general have a positive correlation. Lehmann-Hasemeyer and Neumayer (2019) confirm the correlation. Uncertainty avoidance can be combined with information-based explanations. In uncertain and turbulent times (measured by market volatility) the informational advantage of local and domestic investments is worth more, because it can be harder to gather valid information during crisis. Consequently, investors are more biased towards domestic and local assets in times of high market volatility, retracting to the familiar and to the local investments is a plausible explanation (Giannetti and Laeven 2016). Overall, uncertainty is always existing, though at varying degrees. Investors try to hedge against uncertainty, i. e. hedging against real exchange rate volatility, deviations from purchasing power parity (PPP), inflation risk and general market volatility. Exhibiting home bias is one solution to hedge.

The real exchange rate is subject to fluctuations and therefore represents uncertainty. That is why investors want to hedge against the variation in the real exchange rate. Since within a common currency area there is no need for exchange rate hedging, local bias cannot be influenced by hedging real exchange rate volatility. In a two-country equilibrium model, distribution costs lead to international price differences and real exchange rate fluctuations. Investors try to hedge against real exchange rate risk by exhibiting home bias (Harms et al. 2015). Michaelides (2003) also shows that the exchange rate volatility has a positive impact on home bias. Challenging these findings, hedging motives may be rejected for explaining home bias when saying that “hedging against price uncertainty is neither necessary nor sufficient for home asset preference” (Eldor et al. 1988, p. 165). However, all of these three studies are model-based and not entirely underlined by empirical evidence.

Fidora et al. (2007) give empirical evidence and acts in favour of Harms et al. (2015) confirming the hedging motive against real exchange rate volatility. Changes in the real exchange rate do have a positive relation to home bias. The impact is higher for home bias in bonds because bond returns are a priori less volatile than returns on equity, thus the volatility of the real exchange rate can influence stronger. Fidora et al. (2007) show that if the real exchange rate volatility is set to zero, home bias in bonds can be reduced by 60% points whereas equity home bias decreases only by about 20% points.

Real exchange rate hedging is related to hedging PPP deviations and inflation risks. With incorporating both inflation risk and deviations from PPP in their model, Adler and Dumas (1983) find that people in different countries hold divergent portfolios. The difference should be able to hedge the inflation risk. The model of Stulz (1981b) also acts in favour of these explanations that investors desire a hedge against PPP deviations and inflation risk. However, Cooper and Kaplanis (1994) challenge the main result of the model of Adler and Dumas (1983). They test the model with empirical data from eight developed markets (i. a. the UK, the U.S., Germany, Japan). Hedging just would be a possible reason if there is a negative correlation between equity returns and domestic inflation and if investors have a very low level of risk aversion which is typically not assumed in real economy (Cooper and Kaplanis 1994). Hedging (of inflation risk) can just be attributed as a reason for home bias if risk aversions are set at a very low level in various models (e.g. model of Adler and Dumas (1983) and Stulz (1981a, b)). In contrast to these theoretical considerations and with conventionally assumed levels of risk aversion, Uppal (1993) even shows that investors should prefer foreign investments in such a setting. Also, Mishra (2015) finds no significant correlation between inflation and home bias. Glassman and Riddick (2001) claim that, with a slightly different approach and a relaxation of the PPP assumption, some of the home bias by Cooper and Kaplanis (1994) can be explained.

Additionally, some other hedging motives are considered to explain the home bias. Stockman and Dellas (1989) show model-based that hedging against price uncertainty of nontraded goods induces home bias. Tesar and Werner (1995) show for five OECD countries that holding a disproportionate number of domestic assets can serve as a hedge against shocks to domestic income which have to happen frequent and at a strong extent. The possibility of hedging human capital risks is also incorporated into some models and examined empirically (Baxter et al. 1998; Coën 2001). The large part of studies finds a positive correlation between returns on domestic assets and human capital.^9^ Therefore, using human capital as a hedge is not reasonable as both variables show a positive and no reciprocal correlation.

Overall, the evidence on hedging motives is discussed controversially in the literature. Some studies are supporting different hedging motives as an explanation for home bias, others cast doubt on the impact of hedging. The evidence that hedging real exchange rate risk is part of an explanation of home bias seems strongest. Since most of the literature about hedging is older than 20 years, examining hedging motives should be subject for further analyses nowadays, especially with respect to newly perceived uncertainties induced by the coronavirus pandemic.

## Implications of home and local bias

Since home bias and local bias do not follow standard portfolio selection theory and CAPM, they have economic implications. Apart from implications at the microeconomic level (differences in performance and returns of individual investors’ portfolios), there is also an impact on a macroeconomic level.

### The performance of biased portfolios

The most-often analysed implications of home bias focus on investment performance. Numerous studies examine explicitly the relation between home bias respectively local bias and the performance of such portfolios. While there is also research that focus on the relationship of concentration of portfolios and performance, it has to be clarified that home-biased portfolios are concentrated towards domestic stocks. That is why both research types give a hint about the performance of home- and local-biased portfolios. There is evidence of both better and worse performance of biased and concentrated portfolios compared to well-diversified investment structures. Especially if information-related causes drive the portfolio bias, a superior performance can be observed.

Shukla and van Inwegen (1995) report that domestic mutual fund investors (from the U.S.) perform better than foreign investors (from the UK) and are able to generate higher returns. For the German market, Hau (2001) confirms that home German traders perform better than foreigners due to the linguistics barrier (non-German speaking countries). For high frequency trading and whether the investor is located close to the stocks’ firms headquarter the performance is even better (Hau 2001). In the Indonesian market, Dvorak (2005) suggests that local investors from Indonesia with an informational advantage generate superior returns compared to foreign investors in the short- and medium-term. Performance is best when combining local individuals’ informational advantage with the expertise and experience of global brokerages. Especially in the short run and for not internationally listed stocks, domestic investors outperform foreign investors due to informational advantages regarding the Finnish market (Kalev et al. 2008). Massa and Simonov (2006) approve higher returns when holding home-biased portfolios.

For real estate investments as well, Eichholtz et al. (2001) report a better performance when investing in real estate companies domestically. In markets with given information asymmetries, portfolio concentration can have positive results on the performance (Choi et al. 2017; Ivkovic et al. 2008), as information asymmetries lead to superior knowledge about investment opportunities. Based on a model with delegated asset management Dziuda and Mondria (2012) show that fund managers who specialize in the domestic market generate higher returns. Thus, at least to some extent home bias seems to be a rational behaviour to exploit superior knowledge.

Conclusions on performance can also be drawn for local bias by comparing the performance of intra-national investors with each other. Coval and Moskowitz (2001) elaborate a better performance and higher returns for local-biased mutual funds in the U.S. market compared to more diversified funds (2.67% higher returns per year, with 100 km as local). Ivkovic and Weisbenner (2005) confirm the superior performance of local-biased individual investors. On average local investments earn annually 3.2% higher returns compared to non-local investments. Thus, both institutional/professional investors (fund managers) and individual investors (households) are able to exploit local information for performance improvement (Coval and Moskowitz 2001; Ivkovic and Weisbenner 2005).

Baik et al. (2010) also report higher returns of local-biased investments. Performance could be pushed even higher if a local focus was combined with diversification in this certain local area (Bodnaruk 2009). This result is in conflict with Ivkovic et al. (2008) who suppose that it yields highest returns if investors invest locally and, on top, concentrate the local investment on very few stocks. The puzzle can be resolved by claiming that Bodnaruk (2009) assumes a general information advantage of local stocks whereas Ivkovic et al. (2008) suppose that investors should specify on the few local assets about which the superiority of information is highest. The performance is also better for local-biased venture capital investments (Cumming and Dai 2010). During uncertain times and a high market volatility, exhibiting local and home bias seems to result in a better performance compared to a higher diversification (Giannetti and Laeven 2016). These findings suggest that exhibiting home and local bias is not necessarily an irrational behaviour as the returns of biased and concentrated portfolios are often higher.

If biased portfolios do not perform better (or even worse) than more diversified portfolios, local bias and home bias should be considered as critical behavioural patterns. Pool et al. (2012) claim that the preference for home-state investments of professional investors in the U.S. does not bring higher returns, especially compared to local investments, even though local (geographically) investments perform better. Loyalty-based home bias in portfolio choice of retirement plans of individual U.S. investors makes up about 20% loss in retirement income (Cohen 2009). For local bias, Seasholes and Zhu (2010) document that biased ‘holding portfolios’ of individual investors do not obtain excess returns. When considering the transaction-based local-biased portfolios the purchase of stocks even underperforms the sale. Huberman (2001) also approves the fact that local bias is not a smart behaviour due to the lack of return improvements.

Relating to the performance of home-biased portfolios, Grinblatt (2000) examines investments in Finland, distinguishes sophisticated investors from unsophisticated investors and shows that foreign investors perform better than any domestic investors. Morse and Shive (2011) support the worse performance theory. Bailey et al. (2007, p. 1) also observe a better “information-processing ability” of foreigners compared to locals resulting in a better performance of foreigners. The economic home bias (EHB) by Levy (2017) shows explicitly the economic loss induced by home bias and is dependent on the correlations of the different markets. If markets are highly correlated, the EHB seems to become insignificant. When neutralizing the means and variances in their calculations, the EHB is significant for the U.S. and France, concluding that the home bias remains a puzzle, because there are no benefits from biased portfolios, rather return losses (Levy 2017).

Overall, the results on performance implications are analogue to the number of findings on the particular reasons. The major part of studied reasons is about information asymmetries. It seems that the reasons that are assumed for local and home bias play a crucial role when determining whether the considered biased portfolio performs better or worse than a well-diversified portfolio. This contributes to the assumptions that it has to be a high-level informational advantage which make investors bias their portfolio in order to exploit the superior knowledge and to obtain higher returns. If home and local bias are driven by informational advantages the results challenge the idea of efficient capital markets and induce implications on the macroeconomic level.

### Further implications at the macroeconomic level

Implications at portfolio and individual level are resulting in performance divergences. But there are also implications of home and local bias at the macroeconomic level, hence concerning the entire economy. Home bias seems to have direct implications on the cost of capital in the considered country, because home bias leads to an inappropriate risk-sharing (Lau et al. 2010). The degree of home bias and the cost of capital have a positive correlation. Besides, in a country with home bias, the trade balance is more sensitive to economic shocks.^10^ Put differently, if investors exhibit little diversification (low risk sharing), new shocks will have a higher impact on the trade balance (Fratzscher and Straub 2013). For Germany, Jacobs and Weber (2012) observe that the local bias also impacts at firm level. The main finding is that stocks of firms located in a holiday region^11^ are traded less on the particular holiday. Normally, locally biased investors would trade the stock but, on a holiday, these local investors show inattention and negligence to the stock market and therefore trade less. The reduction in trading at aggregated stock level is significant and observable and confirms strong local bias (Jacobs and Weber 2012). Differences in information release are rejected as an explanation.

An influence of home or local bias on the cost of capital, trade balance sensitivity and stock turnover rates can be assumed. However, as the findings are limited to just a few studies, the macroeconomic implications of home and local bias should be subject to future research.

## Conclusion

This review summarizes the state of the literature on home and local bias. There is much empirical evidence on the existence of home bias and local bias. Investors do not diversify according to standard CAPM. This behaviour is observed for many countries, various asset types and both individual and professional investors. However, the degree varies across time and country. The degree has been decreasing, mainly due to relaxing capital controls and by eliminating explicit barriers (transaction costs) to foreign investments. Though, since there is no unified approach of how to measure the extent of home bias, the results vary notably, especially across countries. No final conclusion on the relation between country and extent can be made.

A large part of the existing literature studies the reasons for home and local bias. Home and local bias are generally considered simultaneously, because the reasons do not differentiate that much. Unfortunately, research on home and in particular local bias is heavily concentrated on U.S. data.

The reasons can be divided into three main categories: institutional reasons, information-based reasons and behavioural/individual reasons. All three categories offer their parts to explain the extent of the bias. This is probably due to various cross-dependencies in economy and also amongst the identified reasons for the bias. In order to understand whether home and local bias are smart, rational or critical behaviours, the performance implications are reviewed. There is much evidence of a better performance of biased portfolios, in particular for those (articles) which also show informational advantages as a reason. However, some research also reports that foreigners perform better while not referring to information asymmetries. Overall, this mixed evidence does not allow to derive recommendations for financial advisors to actively push clients to more internationally diversified portfolios.

The review gives some avenues to future research. Table 2 shows an overview and summarizes them. First, home bias seems to be declining over time, but the decrease has not been researched intensively. The variation in the degree during a course of time and in various countries is still not explained convincingly and should be examined in time series studies. Additionally, there is still no standardized and integrated method of measuring the bias. On top of that, the empirical research should be more thoroughly elaborated for countries beyond the U.S. For example, could future studies select certain measurements of home bias and elaborate the development over time, comparing them with the U.S. results. Second, it should be studied whether ambiguity aversion causes or has implications on local bias, as well the relationship between ambiguity aversion of institutional investors and home bias/local bias might be examined. Third, as some authors indicate that financially literate investors show a better diversification in their portfolios financial literacy (in combination to the probability of advice seeking) is presumably influencing home and local bias as well. As far as we are informed, there are no studies examining this relationship which seems to be a promising avenue for explaining a part of the home bias. Fourth, French (2008) points out the influence of the digitalization and technology progress on trading costs and shows that an overall decrease can be observed. Therefore, even though a decrease of home bias can be assumed over time, the influence of recent technology advancement, as for example algorithmic trading or robo advisors, on asset allocation should be studied more thoroughly. Fifth, since the evidence in this area is very limited, the implications of home bias and local bias have to be subject to further research, in particular using new and broader data. The findings presented in this study are not based on substantial numerous evidence but rather on singular studies.

**Table 2:** Identifying research directions in home bias research

Category	Gap/issue	Research direction
Home bias in the course of time	The potential decrease of home bias has not been studied intensively for various countries	Develop a time series study examining home bias in various countries.
Measurement of home bias	No standardized measurement of home bias	Develop an integrated and standardized measurement of home bias and apply it to various countries
Home bias of various countries	Existing studies focus only on the US	Analyzing reasons for home bias country-specifically
Reasons for home bias	The relation between home bias and ambiguity aversion (as well as financial literacy) is just brought up but not subject to elaborate research	Study these two reasons more detailed in order to find a potential causal relationship between ambiguity aversion (and financial literacy) and home bias
Factors influencing home bias	There are no studies elaborating the impact of recent developments in technology and digitalization on home bias	Study the relationship between digitalization and trading costs and consequently, a potential decrease of home bias
Implications of home bias	The implications of home bias are not very thoroughly studied and evidence is limited	Use new and broad data for different countries to examine (yet unresearched) implications of home bias
Literature selection	Research is mainly based on peer-reviewed articles	Grey literature should be scanned if results are substantial or provide future research avenues

Apart from the limitations regarding home bias’ implications, one further limitation of the review is incorporating all relevant studies. As indicated with the full-text search of Google Scholar there are numerous results when searching for home and local bias. It is impossible to achieve a fully comprehensive review of all studies dealing with home bias since the field is very broad. Some studies which even not explicitly elaborate home bias may be suited for explaining at least a part of home bias. However, it should always be taken into account that the differentiation between correlations and causalities in the considered studies is crucial. The absolute difference in cited studies compared to the literature reviews of both Coeurdacier and Rey (2013) and Ardalan (2019) strengthens the limitation. Apart from the described limitations of the method of literature search, it has to be mentioned again that all types of grey literature are not included in this review. Despite the benefits of this procedure that thereby just peer-reviewed articles are included, the exclusion of grey literature also constitutes a limitation of this review and could change the structure of this review considerably (Fisch and Block 2018).

## Appendix

See Table 3.

**Table 3:** Number of findings in the journal databases and other sources with different search requests

	Advanced			Full-text	Date of search
	Home bias	Local bias	Domestic bias	Home bias/home bias	
*Journal*					
The Journal of Finance	2	1	1	468/70	6th May 2019
Journal of Financial Economics	11	3	0	366/58	6th May 2019
The Review of Financial Studies	3	0	0	306/17	6th May 2019
Journal of Financial and Quantitative Analysis	2	0	0	124/87	6th May 2019
Review of Finance	3	2	0	139/63	6th May 2019
Journal of Banking & Finance	22	3	0	595/114	6th May 2019
Journal of Economic Dynamics & Control	7	0	0	151/67	6th May 2019
Journal of Financial Intermediation	0	0	0	62/4	7th May 2019
Journal of Money, Credit and Banking	3	0	0	152/37	7th May 2019
Review of Derivatives Research	–	–	–	1	7th May 2019
*Source*					
Library catalogue of Technical University of Darmstadt	26	1	0	77	6th June 2019
Google Scholar	980	190	28	25.600/3.940.000	6th June 2019

## Footnotes

1 The online journal databases of all ‘A+’- and ‘A’-rated journals of the VHB-ranking (‘Banking & Finance’; ‘General Business Management’) are searched.

2 Subjective is used in the meaning of not measurable. For a rating of a specific individual, this is the case. Since the ratings of all the individuals are calculated together, the VHB-ranking also has some objective traits.

3 These two journals are not included in any of the Scimago impact factor surveys.

4 For example, Coval and Moskowitz (2001) define a 100-km-radius as local.

5 Relating to papers starting from 2004.

6 The findings of Lütje and Menkhoff (2007) are just valid for equity managers and not for bond managers.

7 Canadian firms are not part of the Non-U.S. sample.

8 Competence hypothesis is based on three pillars: First there is a preference for betting on the known compared to the unknown. Second, a preference to bet on future rather than on past events. Third, a preference for skill-based betting compared to pure chance (Heath and Tversky 1991).

9 Only Bottazzi et al. (1996) find a negative correlation between human capital and domestic securities.

10 News shocks can be seen amongst others as an “increase in domestic equity prices relative to the rest of the world” (Fratzscher and Straub 2013, p. 1211).

11 Germany is divided in 16 states. Some holidays are only holidays in some states and in others not. A holiday region is a region in which a holiday is official whereas in a non-holiday-region a holiday cannot be applied (Jacobs and Weber 2012).
